# Coronavirus Disease Exposure and Spread from Nightclubs, South Korea

**DOI:** 10.3201/eid2610.202573

**Published:** 2020-10

**Authors:** Cho Ryok Kang, Jin Yong Lee, Yoojin Park, In Sil Huh, Hyon Jeen Ham, Jin Kyeong Han, Jung Il Kim, Baeg Ju Na, Seoul Metropolitan Government COVID-19, Rapid Response Team (SCoRR Team)

**Affiliations:** Seoul Metropolitan Government, Seoul, South Korea (C.R. Kang, H.J. Ham, J.K. Han, J.I. Kim, B.J. Na);; Seoul National University Boramae Medical Centre, Seoul (J.Y. Lee);; Seoul Centre for Infectious Disease Control and Prevention, Seoul (Y. Park, I.S. Huh)

**Keywords:** respiratory infections, severe acute respiratory syndrome coronavirus 2, SARS-CoV-2, SARS, COVID-19, 2019 novel coronavirus disease, coronavirus disease, zoonoses, viruses, coronavirus, outbreak, nightclubs, Seoul, South Korea

## Abstract

At least 246 cases of coronavirus disease (COVID-19) have been linked to nightclubs in Seoul, South Korea. During the April 30–May 5 holiday, young adults from across the country who visited nightclubs in Seoul contracted COVID-19 and spread it nationally. Nightclubs were temporarily closed to limit COVID-19 spread.

South Korea had 10,801 confirmed cases of coronavirus disease (COVID-19) by May 4, 2020 (*1*). The epidemic curve of the cumulative number of cases had plateaued in April (Appendix). Nightclubs that had been closed as part of the social distancing policy reopened on April 30, ahead of the April 30–May 5 Golden Week holiday. People from around the country visited the Itaewon area (Itaewon-dong) in downtown Seoul during the holiday period. Itaewon is known for its diversity and contains a US Army base, multiple embassies, and several well-known nightclubs.

Starting on May 6, several COVID-19 cases were confirmed among persons who had visited nightclubs in Itaewon during the holiday. Secondary transmission by case-patients linked to the Itaewon nightclubs led to local transmission of COVID-19 in other parts of the country (Figure). On May 9, the Seoul Metropolitan Government announced indefinite closure of all nightclubs in Seoul to control the source of the outbreak. Subsequently, several regions prohibited mass gatherings.

**Figure:**
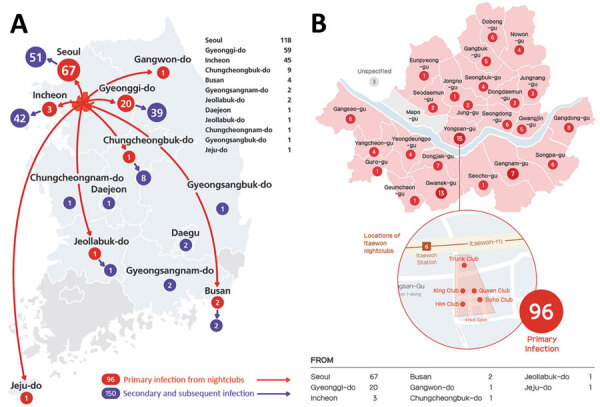
Cases related to the COVID-19 outbreak in nightclubs in Itaewon, Seoul, South Korea, that were diagnosed in major cities and provinces of South Korea as of May 25, 2020. A) Distribution of cases by city (n = 246). B) Distribution of primary and secondary cases contracted in nightclubs within the Seoul metropolitan area, by **neighborhood** in which the nightclubs are located (n = 118, of which 96 contracted the disease in Seoul nightclubs).

The Seoul Metropolitan Government and Yongsan-gu Office, in cooperation with the Seoul Metropolitan Police Agency, conducted contact tracing of persons who had visited any of the 5 major nightclubs in Itaewon during April 30–May 6. The use of cell phone location data, credit card records, and lists of nightclub visitors led to the identification of 5,517 persons for screening; of those, 1,257 were actively monitored. An additional 57,536 persons who had spent >30 minutes in the vicinity of the nightclubs, as determined by their cell phone location data, were sent a series of text messages encouraging them to undergo testing.

After media outlets reported that venues at the epicenter of the outbreak were gay nightclubs, a rumor spread that this COVID-19 outbreak originated among gay men. Authorities became concerned that this rumor could adversely affect nightclub visitors’ willingness to be tested. Because of prejudice against homosexuality, gay men in South Korea usually experience discrimination and stigmatization and so are often unwilling to reveal their sexual identity (*2*). Thus, the Seoul Metropolitan Government consulted sexual-minority groups to discuss ways to encourage testing among gay men. The sexual-minority groups recommended anonymous testing. Therefore, the Seoul Metropolitan Government introduced anonymous testing and stated that the only information that patients were required to provide was their cell phone number for contact purposes. Through the lesbian, gay, bisexual, and transgender community, we advertised that screening clinics of public health centers were conducting anonymous testing for COVID-19; we also advertised anonymous testing through mass media. 

We conducted large-scale testing for active case-finding among persons who had visited the Itaewon nightclubs. Patients’ cell phone numbers were checked on site before testing. Demographic data were obtained by contacting those who tested positive. Of the 41,612 total tests conducted by May 25, a total of 35,827 (86.1%) were conducted on Itaewon nightclub visitors, 5,785 (13.9%) on contacts of case-patients linked to the Itaewon nightclubs, and 1,627 (3.9%) tests conducted on anonymous persons. The prevalence of positive results for COVID-19 in nightclub visitors was 0.19% (67/35,827); in their contacts, 0.88% (51/5,785); and in anonymously tested persons, 0.06% (1/1,627).

As of May 25, a total of 246 confirmed nightclub-associated cases had been reported; 96 (39%) of those were primary cases and 150 (61%) were secondary cases (Figure). The estimated attack rate among nightclub visitors was 1.74% (96/5,517). Of the total number of confirmed cases, 118 positive case-patients (47.9%) live in Seoul; among those, 67 (56.8%) were primary cases, 32 (27.1%) secondary cases, 7 (5.9%) tertiary cases, 4 (3.4%) quaternary cases, 4 (3.4%) fifth-order cases, and 4 (3.4%) sixth-order cases. Infections related to the nightclub outbreak continued to spread further in the community; in Seoul, COVID-19 cases related to the outbreak were identified in 9 different workplaces (several companies, the Army base, and a hospital) and 6 multiuse facilities (pubs, coin karaoke facilities, and a fitness center). In addition, we identified 7 cases of household transmission (Appendix).

In summary, we identified 246 COVID-19 cases associated with the reopening of nightclubs in Seoul. To conduct contact tracing for this outbreak, we used multiple forms of advanced information technology, including location data from mobile devices, credit card payment history, geographic positioning service data, drug utilization review, public transportation transit pass records, and closed-circuit television footage (*3*). Despite the low incidence of COVID-19 in the postpeak period of the pandemic, superspreading related to visiting nightclubs in Seoul has the potential to spark a resurgence of cases in South Korea.

Appendix Additional information about coronavirus exposure and spread from nightclubs in Seoul, South Korea.
