# Supplementary material for: Coronavirus Disease Exposure and Spread from Nightclubs, South Korea
Source: Emerg Infect Dis. 2020 Oct;26(10):2499–501. doi: 10.3201/eid2610.202573 (PMC7510694; doi:10.3201/eid2610.202573)
Supplement: Appendix — Additional information about coronavirus exposure and spread from nightclubs in Seoul, South Korea. [file 20-2573-Techapp-s1.pdf]

[Article DOI: https://doi.org/10.3201/eid2610.202573](https://doi.org/10.3201/eid2610.202573)

Coronavirus Disease Exposure and Spread from Nightclubs, South Korea

Appendix

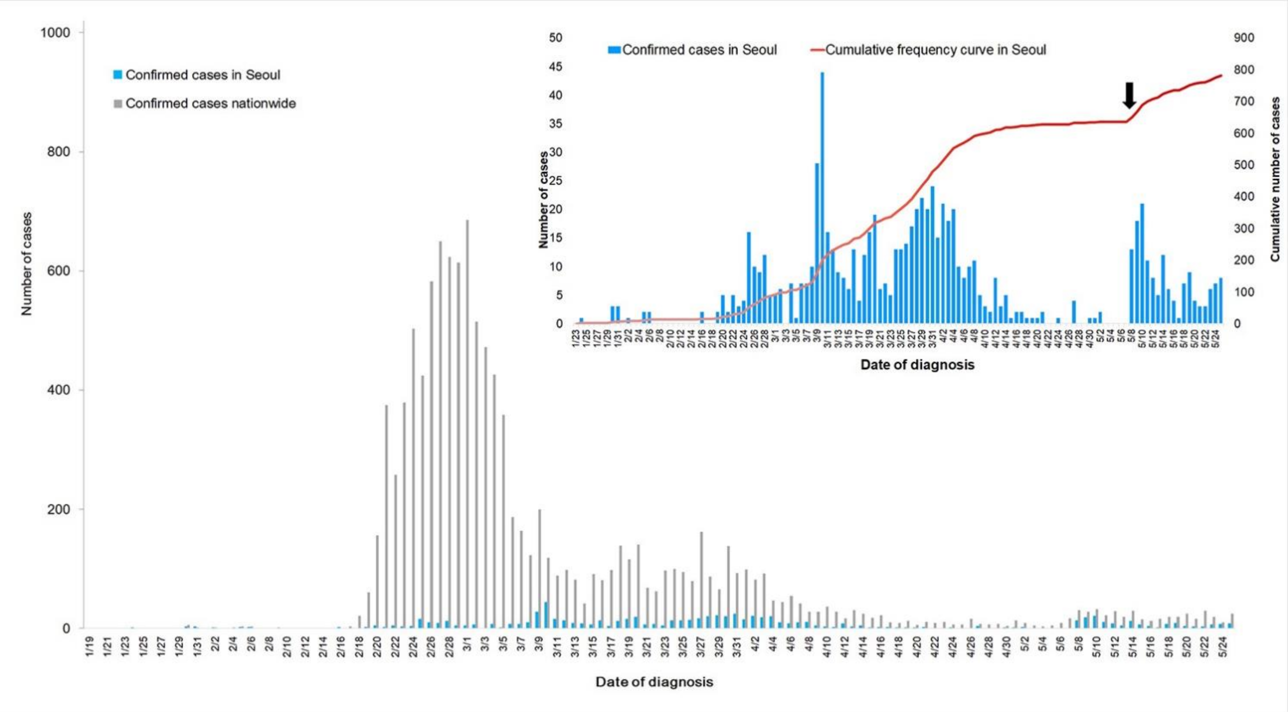

Appendix Figure 1. A) Epidemic curves of confirmed cases of coronavirus disease in Seoul, South Korea, and nationally through May 25, 2020. B) Cumulative cases and frequency curve of coronavirus disease in Seoul.

-

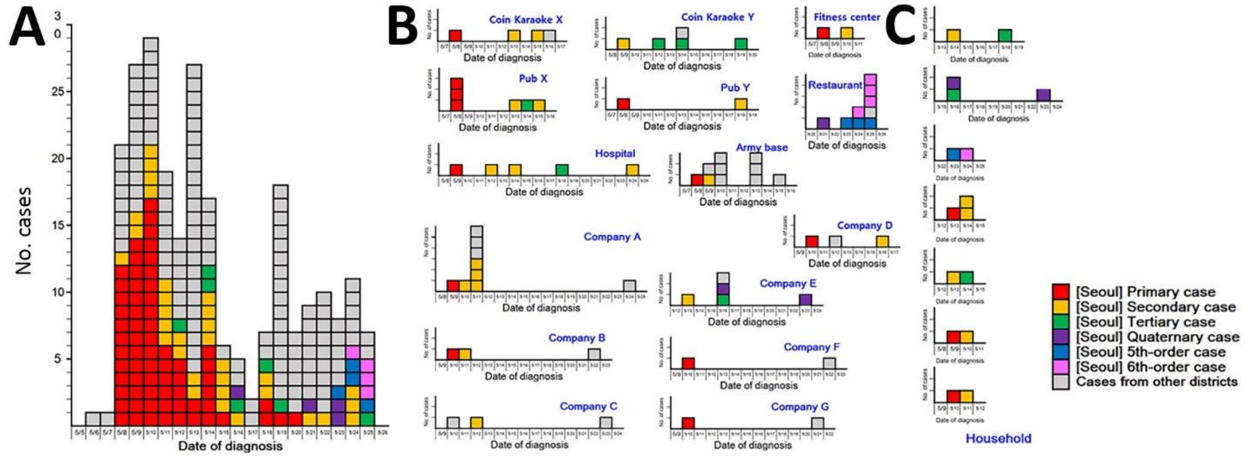

Appendix Figure 2. Epidemic curves of the COVID-19 outbreak related to nightclubs in Itaewon, Seoul, as of May 25, 2020. A) Overall epidemic curve of the COVID-19 outbreak related to Itaewon nightclubs. B) Epidemic curves of the COVID-19 cases by facility with an outbreak. C) Epidemic curves of COVID-19 household transmission by persons related to Itaewon nightclub visits in Seoul.
